# Development of a Dye-Based Device to Assess the Poultry Meat Spoilage. Part II: Array on Act

**DOI:** 10.1021/acs.jafc.0c03771

**Published:** 2020-10-29

**Authors:** Lisa Rita Magnaghi, Giancarla Alberti, Federica Capone, Camilla Zanoni, Barbara Mannucci, Paolo Quadrelli, Raffaela Biesuz

**Affiliations:** † Department of Chemistry, University of Pavia, Via Taramelli 12, 27100 Pavia, Italy; ‡ Centro Grandi Strumenti, University of Pavia, Via Bassi 21, 27100 Pavia, Italy; § Unità di Ricerca di Pavia, INSTM, Via G. Giusti 9, 50121 Firenze, Italy

**Keywords:** dyes, sensors array, meat spoilage, naked-eye detection, real chicken samples, BAs do not fly

## Abstract

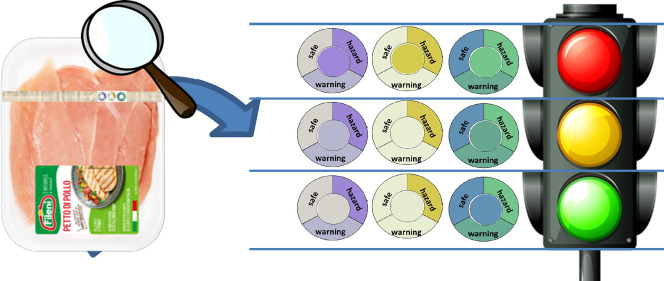

This work presents a colorimetric dye-based array for naked-eye detection of chicken meat spoilage. The array is obtained by fixing five acid–base indicators, *m*-cresol purple (**1**), *o*-cresol red (**2**), bromothymol blue (**3**), thymol blue (**4**), and chlorophenol red (**5**), and a sensing molecule specific for thiols, 5,5′-dithiobis(2-nitrodibenzoic acid), called Ellman’s reagent (**6**), on a cellulose-based support. The dyes, being permanently charged, are fixed on the support via ion-exchange. The entire degradation process of beast poultry meat, at ambient temperature and in a domestic fridge, is followed by the change of the color of the array, placed in the headspace over the meat samples. The device is set after selection of the most suitable starting form, which could be the acidic or the basic color of indicators, being the proper dye concentration and the dimension of the spots already established. Basing on sensors colors, we identified three levels of the degradation process of chicken meat, named SAFE, WARNING, and HAZARD. By instrumental analysis, we demonstrated that sensors response was correlated to volatile organic compounds (VOCs) composition in the headspace and, thus, to meat spoilage progress. We demonstrated that biogenic amines (BAs), commonly considered a critical spoilage marker, are indeed produced into the samples but never present in the headspace, even in traces, during the investigated time-lapse. The VOC evolution nevertheless allows one to assign the sample as WARNING and further HAZARD. Some indicators turned out to be more informative than others, and the best candidates for a future industrial application resulted in a bromothymol blue (**3**)-, chlorophenol red (**5**)-, and Ellman’s reagent (**6**)-based array.

## Introduction

Meat spoilage is a very complex combination of processes, related to the activity of different bacteria, which, depending on external conditions, are responsible for the oxidation of glucose, lactic acid, and fatty acid and, eventually, the degradation of proteins.^1,2^

Commonly, the methods employed to evaluate meat quality require instrumental or microbiological analyses and sensory evaluation. They are all destructive and expensive; they require specialized people and have a long-time response.

Numerous efforts are underway to develop automated techniques and/or methods that allow continuous and simple monitoring for in-field application, like home setting, supermarket, and stores. In this way, through simple, low-cost, and highly efficient and effective methods, also consumers could directly detect the meat freshness.^1^ We can include in this category biosensors, electronic devices (e-nose and e-tongue),^2^ and colorimetric sensors.

Colorimetric-based sensors are capable of changing color to a reaction with volatile compounds produced in the headspace of packaged meats. Either included directly on packaging or attached with an on-package sticker, they offer the simplest, practical, instrument-less way for monitoring meat freshness, directly by the naked eye.

The recent literature presents many examples that follow this idea, for instance, immobilized bromocresol green as a fish spoilage indicator or mixed pH dye-based indicator as a “chemical barcode” (using bromothymol blue and methyl red or bromothymol blue, bromocresol green and phenol red as a single indicator).^1^ The disadvantage with a single sensor is similar to that encountered in a classical acid–base titration technique using a pH dye. It is difficult to determine the onset of detection related to the spoilage threshold, where it could be too early or too late when correlated with microbial growth.

It must be underlined that, in the literature, the attention in papers dealing with chromogenic sensors was always focused on the production and identification BAs, not only into the meat but also in the headspace.^3−6^ Even in the most recent literature. the focus is still on the developments of free BAs.^7,8^ Two aspects have to be pointed out. First, in the early spoilage, other catabolic reactions take place.^9^ The chemical spoilage index (CSI) is associated with the consumption of glucose and lactic acid and production of EtOH, 3-methyl-1-butanol, and free fatty acids, mainly acetic acid,^9^ which are definitively the dominant VOCs. Any meat at this stage is still a safe product, and in the development of a proper sensing device, its ability to recognize this stage must not be neglected.

Only when no more glucose and none of its direct metabolites are left, the catabolism of proteins starts, and the production of amines and thiols is manifested as off-odors and discolouration.^9^ Due to the toxicity of these classes of byproducts,^10^ consumption of meat at this stage could be a severe hazard. For this reason, the presence of amines (putrescine, cadaverine, histamine, tyramine, spermidine, spermine, and ethylamine) is an important indicator of food quality and hygiene.^10^

Second, BAs are produced into meat samples, but they have never been detected in the headspace, where sensing devices are usually included. This aspect can be explained considering that BAs are weak bases and are involved in one or more protonation equilibria between species with a different charge. Since only neutral molecules can fly, their volatility depends on the pH of the medium. In the case of meat and other biological matrices, the pH is buffered^11^ at a value around neutrality at which the amines are present in their positively charged protonated form, as will be further discussed in the text below , and thus cannot be present in the headspace. In this first attempt, we limited our attention to chicken meat for its high perishability and large diffusion.

With this in our mind, we present a dye-based colorimetric array selecting five different dyes that change their color in a pH range around neutrality.^12,13^ We want to stress that the pH interval of interest is more limited than expected if BAs were present, and it does not make any sense to explore a broad pH region, as done by other researchers.^14^ Furthermore, we chose to develop the array directly on real samples, not on enriched amine samples^4^ or samples left moldering for weeks^15^ since, under these circumstances, a consumer definitively does not need any sensor to assess the stage of spoilage.

These aspects make the difference with the existing colorimetric sensors. We will demonstrate that the evolution of the array’s colors follows the entire spoilage process from the very beginning to the end of the SAFE condition, going to a WARNING period until the HAZARD one.

The simplified version of the array (with bromothymol blue (**3**), chlorophenol red (**5**), and Ellman’s reagent (**6**) sensors) is based on simple and cheap reactions and reagents. We demonstrated that the device gives reliable, individual, easy to interpret information, being developed, tested, and validated on real samples, under usual consumers’ conditions.

In our idea, this study is the base for developing an intelligent label. Such a label accounts, on one hand, for improper meat conservation or treatment as it might happen after the shopping, but at the same time, it can ensure a safe consumption beyond a use-by date since it gives information on the spoilage state of that individual tray. Such a kind of label suggests to the end-user, at home, how and if it is safe eating a piece of meat found in the bottom of the fridge. The consumer can do it merely by a naked-eye evaluation, without any instrument, without any app, as already proposed.^2,16^ The industrial application of such a device could be a future scenario due to the robust reliability and the very reduced costs in a label implementation. A patent based on this idea has been deposited^17^ and more recently the extension to WIPO PCT.^18^

## Materials and Methods

All reagents were of analytical reagent grade. *m*-Cresol purple (**1**), *o*-cresol red (**2**), bromothymol blue (**3**), thymol blue (**4**), chlorophenol red (**5**), and Ellman’s reagent (**6**) were purchased by Carlo Erba or Sigma Aldrich.

Dylon Colour Catcher (CC) was bought in a local supermarket.

Beast poultry meat in slices was bought in a local supermarket (UNES Supermarkets, via Fratelli Cervi, 11 27100 Pavia), the same day of the delivery from the central slaughterhouse few moments after the meat was put on the shelf.

Pictures of the array were taken by a Smartphone Samsung Galaxy S7; a portable LED light box was used to guarantee the reproducibility of the photos (PULUZ, Photography Light Box, Shenzhen Puluz Technology Limited).

The setup and the analytical performance of the array were already discussed in the first part, see reference.^19^ The usage of CC as a solid support was also reported previously^20,21^ where Alizarine Red S and the Ellman’s reagent were employed as the receptor of two different sensors.

### The Chameleon Array

The dyes selected for the array are six, see the first part, see reference.^19^ The first five, *m*-cresol purple (**1**) (log *K*_a1_ = 8.32, log *K*_a2_ = 1.57), *o*-cresol red (**2**) (log *K*_a1_ = 8.20, log *K*_a2_ = 1.11), bromothymol blue (**3**) (log *K*_a_ = 7.1), thymol blue (**4**) (log *K*_a1_ = 8.90 log *K*_a2_ = 1.50), chlorophenol red (**5**)( log *K*_a_ = 6.0), are acid–base indicators, with their log *K*_a_ values.^12,13^ The sixth is the 5,5′-dithiobis(2-nitrodibenzoic acid) (**6**), generally called Ellman’s reagent. In the presence of thiols, it readily undergoes a trans-sulforation reaction with the reduction of the sulfhydryl group that releases a highly chromogenic product, 5-thio-2-nitrobenzoate (TNB), with an intense absorption band at 412 nm.^22^ All these molecules present a permanent negative charge, two in the case of Ellman’s reagent. For convenience, in the following, the dyes are ordered from 1 to 6, as reported above.

### Preparation and Experimental Setup for the Final Array

The CC was cut in circles of 0.4 cm in diameter of approximately 0.0015 g, obtained with a hole punch for paper, as described elsewhere in the Introduction together with the final experimental setup.

During the synthesis of each sensor, the exchange reaction between the CC and the receptors turns the dyes into their basic color. Nevertheless, the starting color of each dye is of paramount importance when we move to the real sample. It must be selected as a function of the headspace composition, thinking of which class of molecules we want to reveal at its best.

Table 1 reports the optimized conditions to prepare the final sensors. Four of them are kept into their basic form, adding with care, a small quantity of acid, to reduce the excess of alkalinity (Figure1S of the Supporting Information shows an example of the experiments performed to select the proper excess of acid or base).

**Table 1:** Experimental Conditions for the CC Spot Sensor Preparation

		dye concentration (M)	μL HCl 10^–3^ M	prevalent starting sensor form
**1**	*m*-cresol purple	7 × 10^–6^	20	basic
**2**	*o*-cresol red	4 × 10^–6^	40	basic
**3**	bromothymol blue	9 × 10^–6^	40	basic
**4**	thymol blue	8 × 10^–6^	10	basic
**5**	chlorophenol red	7 × 10^–6^	500	acidic
**6**	Ellman’s reagent	2.4 × 10^–5^	100	

**Figure 1:**
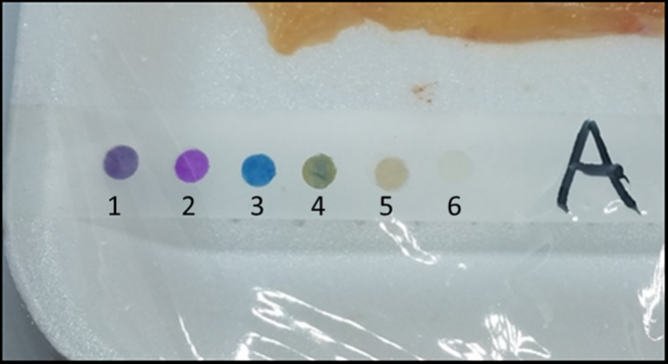
An example of the array placed over the tray containing the poultry meat, with sensors from one to six based on *m*-cresol purple (**1**), *o*-cresol red (**2**), bromothymol blue (**3**), thymol blue (**4**), chlorophenol red (**5**), and the Ellman’s reagent (**6**).

The beast poultry meat was purchased, as already said, in a local supermarket. We bought directly ready trays made of a plastic container (PP) for food and covered with low-permeability polyethylene plastic film. The trays were taken from the shelf, just a few moments after preparation (from previous agreements with the head of the butcher’s department), to ensure a homogeneous lifetime of all samples. Within 10 min, the samples were in the lab, the plastic film was removed, the stripes with sensors were placed over the tray, and a new plastic film was fixed around the tray. The samples were placed under the hood or in the fridge, depending on the type of experiment. Figure 1 shows a picture of the experimental setup.

### Color Analysis

An exhaustive discussion on photo acquisition is found in the first part, see reference.^19^ The photos were acquired by a Smartphone Samsung Galaxy S7 in a lightbox to ensure a constant and reproducible light exposition.

The RGB space color was preferred to others. The GIMP software, an open-source program,^23^ was employed, which allows defining the area of the photo to be analyzed, usually here the entire spot, and gives back the average values of the RGB triplet for each sample.

### Real Sample Analysis

We took pictures, as a function of time, of the array placed in the headspace of five or six different samples of beast poultry meat kept at ambient temperature or in the fridge, depending on the experiment. The corresponding RGB triplets for each sensor were acquired. The principal component analysis, PCA, was performed, centering but not scaling the data since the RBG triplets are intrinsically scaled (RGB values vary from 0 to 255). Even if other chemometric techniques could be employed in this case, we want simply to rationalize the color evolution that at a first look was less intense than expected, and PCA analysis was suitable for this purpose. The open-source program CAT (Chemometric Agile Tool) was employed.^24^

### Samples for Validation

We bought three meat samples at the local supermarket to perform the instrumental validation. The samples were prepared with the array as described above, but for each sample, around 20 g was cut away, under extremely clean conditions, to avoid contaminations. Two subsamples of around 5 g (see below) were inserted into a vial for the meat analysis, and other two of around 5 g were sealed into different vials equipped with the solid phase for the headspace analysis (see below). The subsamples and the tray were kept under the same condition, and the color array was the reference. At a given time, the array was photographed, and the content of the corresponding vials was immediately submitted to analysis of the meat and headspace, as described in the paragraph below. We have an independent meat sample for each degradation step.

### Instrumental Analysis

The instrumental analyses for validation of the different degradation steps were performed at CGS (Centro Grandi Strumenti), which is one of the facilities of Pavia University.

For the analysis of the meat samples, we followed the procedure suggested in the literature.^25^ Each piece of meat, at the given degradation step, was cut in a blender, and 4 g was extracted by Ultra-Turrax S 18N-10G homogenizer (IKA-Werke Gmbh & Co., Germany) and 5% TCA (trichloroacetic acid); after the centrifugation step, the supernatants were collected and purified on SPE STRATA X cartridges (conditioned with 4 mL of methanol followed by 4 mL of Milli-Q water). Then, 2 mL of the sample (supernatant), with a pH adjusted around 11 by adding 200 μL of NH_4_OH 28%, were passed through the cartridges. After complete loading, cartridges were rinsed with 2 mL of a mixture of MeOH/H_2_O (5/95 v/v) and then dried under vacuum to remove the excess of water. Analytes were eluted from the STRATA X sorbents with 2 + 2 mL of a solution of methanol/acetic acid (99/1 v/v). The eluting solution was dried with nitrogen gas, and the residue was collected with 2 mL of 0.1 M HCl, filtered, and injected into the LC-MS/MS (liquid chromatography linked to tandem mass spectrometry).

On the other hand, for the volatile analysis, samples of the vials equipped with the solid phase were analyzed by LC-MS/MS. The analyses were performed directly using the Head Space Solid Phase Microextraction (HSSPME) coupled with gas chromatography–mass spectrometry (GC/MS). The following experimental parameters were used: fiber polydimethylsiloxane/divinylbenzene (PDMS/DVB) = 65 μm; extraction temperature = 35 °C; extraction time = 20 min; desorption temperature = 250 °C; desorption time = 4 min. GC/MS analysis was performed on a Thermo Scientific DSQII single quadrupole GC/MS system (Thermo Fisher Scientific, Milan, Italy) equipped with a Restek Rtx-5 MS capillary column (30 m, 0.25 mm i.d., 0.25 μm film thickness), with helium as a carrier gas at a constant flow rate of 1.0 mL/min. The injector temperature was set at 250 °C, and it was operated in splitless mode. The oven was held at 35 °C for 2 min, and then the temperature was increased to 80 °C at a rate of 5 °C/min, ramped to 300 °C at a rate of 10 °C/min, and finally held at 300 °C for 2 min. The GC transfer line temperature was 270 °C. All mass spectra were acquired in electron impact mode (ionization energy 70 eV, source temperature 250 °C), with spectra acquisition in full scan mode (mass range *m*/*z* 15–650 amu, scan speed = 832 amu/s). Assignment of chemical structures to chromatographic peaks was based on the comparison with the databases for GC/MS NIST Mass Spectral Library (NIST 08) and Wiley Registry of Mass Spectral Data (8th edition). The Xcalibur MS Software version 2.1 (Thermo Scientific Inc.) and the AMDIS Program for the automated deconvolution of mass spectra were used for GC/MS data interpretation.

### Samples for Sensitivity Test

The degradation model, developed according to the color of the different sensors of the array, refers to a well-defined experimental setup. In general, the tray, sealed with the plastic film, contains a volume of about 500 cm^3^, and the slides of beast poultry meat have a mass always around 300 g. Decreasing the amount of the sample, on the same tray, all the analytes dilute progressively; so, it is important to evaluate the sensitivity of the array and estimate the lowest amount of poultry meat that produces an equal color evolution.

For this reason, from the usual amount of meat used for the model development, we prepared samples with a progressively lower meat mass, from 300 g for the first sample, halving each time the amount, to 18.8 g of the fifth sample. We sealed each portion in the common tray and analyzed them following the procedure explained before,

## Results and Discussion

### Evolution of the Colors over the Poultry Meat at Different Temperatures

The setup of the array, to explore its chameleon properties and to assess the meat spoilage, was discussed in the first part, see reference.^19^ The selection of the most suitable acid or base form for each indicator is reported above (see the Materials and Methods section). Here, the evolution of the array color over five different trays of beast poultry meat kept at room temperature was registered.

**Figure 2:**
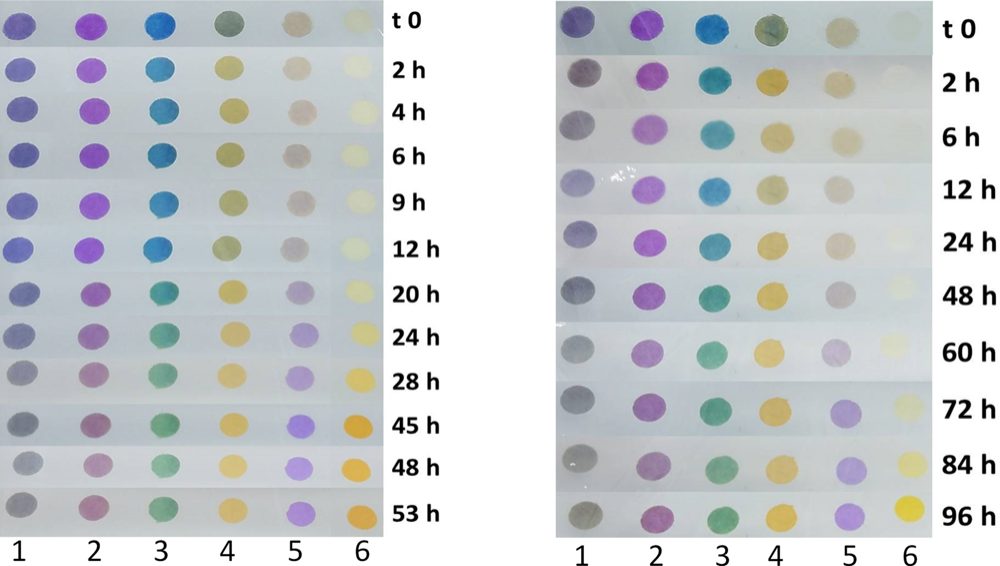
Evolution of the colors of the array (sensors placed in the usual order from 1 to 6) in a headspace of a tray containing poultry meat, registered as a function of time. On the left, the case of a sample of kept out of the fridge, and on the right, the case of another sample kept in the fridge.

At a given time, the tray was put into the lightbox, a photo of the array was acquired, and the RGB triplet of each sensor was registered. As an example, Figure 2, on the left, shows the photos collage of one sample as a function of the time in hours (reported on the right of each picture). The evolution of dyes could also be appreciated by the naked eye even if the meaning is not immediately clear.

**Figure 3:**
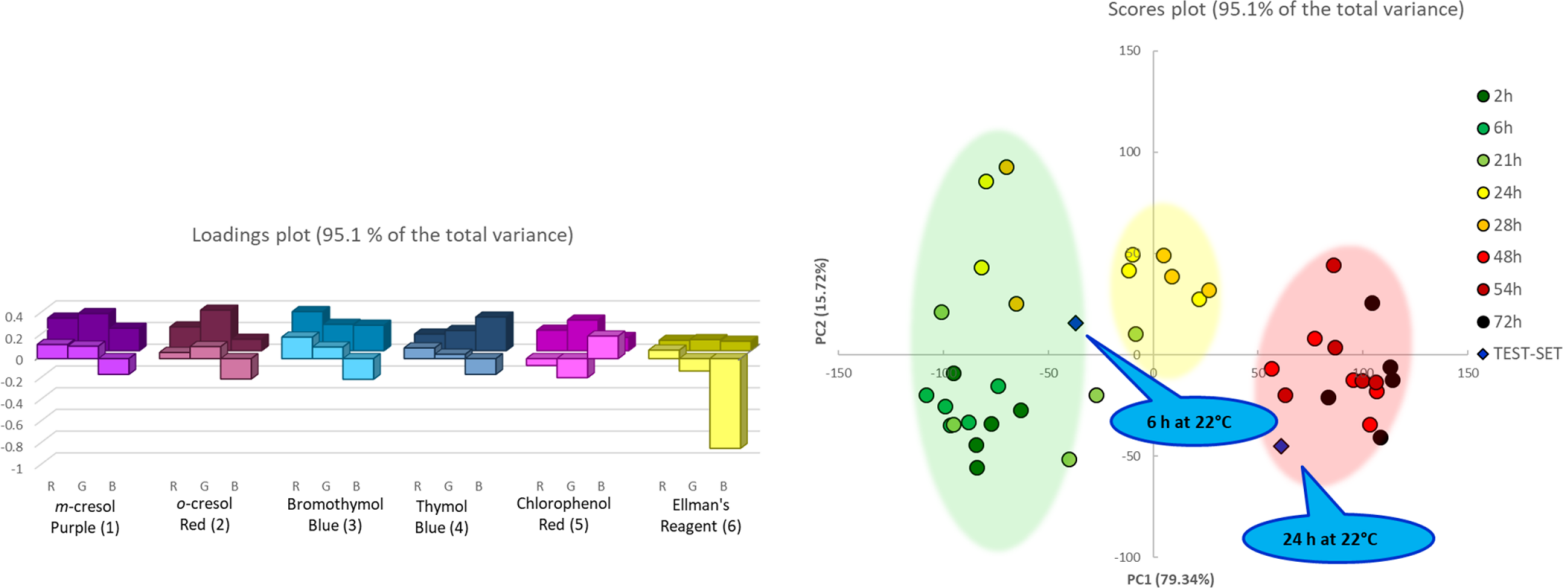
PCA model on five different samples of beast poultry meat for samples left out of the fridge. On the left, the loading plot, in foreground values on the PCA1, in the background those on PCA2. On the right, the score plot. The green, yellow, and red shadow areas collect samples defined SAFE, WARNING, and HAZARD, respectively, exclusively added, here and in the following, as a simplification of the different groups.

The same strategy was applied to the five samples of beast poultry meat, but in this case, we put the trays in a domestic fridge. The photos of one tray are shown in Figure 2, on the right, and for the other samples, in the Supporting Information (Figures 2S and 3S) for both types of conservation.

The two series of photos look similar; they only differ in the time evolution, much more expanded for sample in the fridge than for the one out of the fridge. This aspect makes sense because the bacteria involved in the two cases, in and out of the fridge, are different,^9,26^ but the degradation produces the same substances in similar amounts, primarily depending on the substrate.

Again, we know that, in the early spoilage stage, the production of alcohol and acids occurs. They can be detected by dyes that turn their color from the basic form into the acidic one: in our array dyes from 1 to 4, because they exhibit log *K*_a_ higher than 7. This evolution can be seen in Figure 3 very clearly. Afterward, in the same atmosphere, if free amines were to be developed, we would have expected a re-establishment of the basic form color of the first four dyes, *m*-cresol purple (**1**), *o*-cresol red (**2**), bromothymol blue (**3**), and thymol blue (**4**). However, only the indicator with a lower log *K*_a_ value of 6.0, i.e., chlorophenol red (**5**), placed intentionally in its acidic form, showed an appreciable color change, meaning that the pH slightly increases but not as expected. In the early stage of this study, we overestimated the potential amines production in the headspace. This aspect was never investigated in the existing literature regarding optical sensors developed to test meat quality; it was accepted but not understood. The key point is that any meat is under buffered conditions, so even if BAs developed into the meat, they do not fly since they are in their protonated form. On the other hand, the change in the color of the sixth sensor, Ellman’s reagent (**6**) sensor, clearly says that thiols are present in the headspace, meaning that the catabolism of protein is indeed going on.

The color evolution, described by the RBG data collected from the two series of samples, was submitted to principal component analysis (PCA), as described in the Real Sample Analysis section. We first examined the five samples kept out of the fridge. Figure 3 shows the PCA model on the two first components that explain more than 90% of the total variance. The scores plot clear separates samples according to a timeline that seems to be associated with the degradation stage quite clearly. The separation looks intriguing, but we must prove that it makes sense.

**Figure 4:**
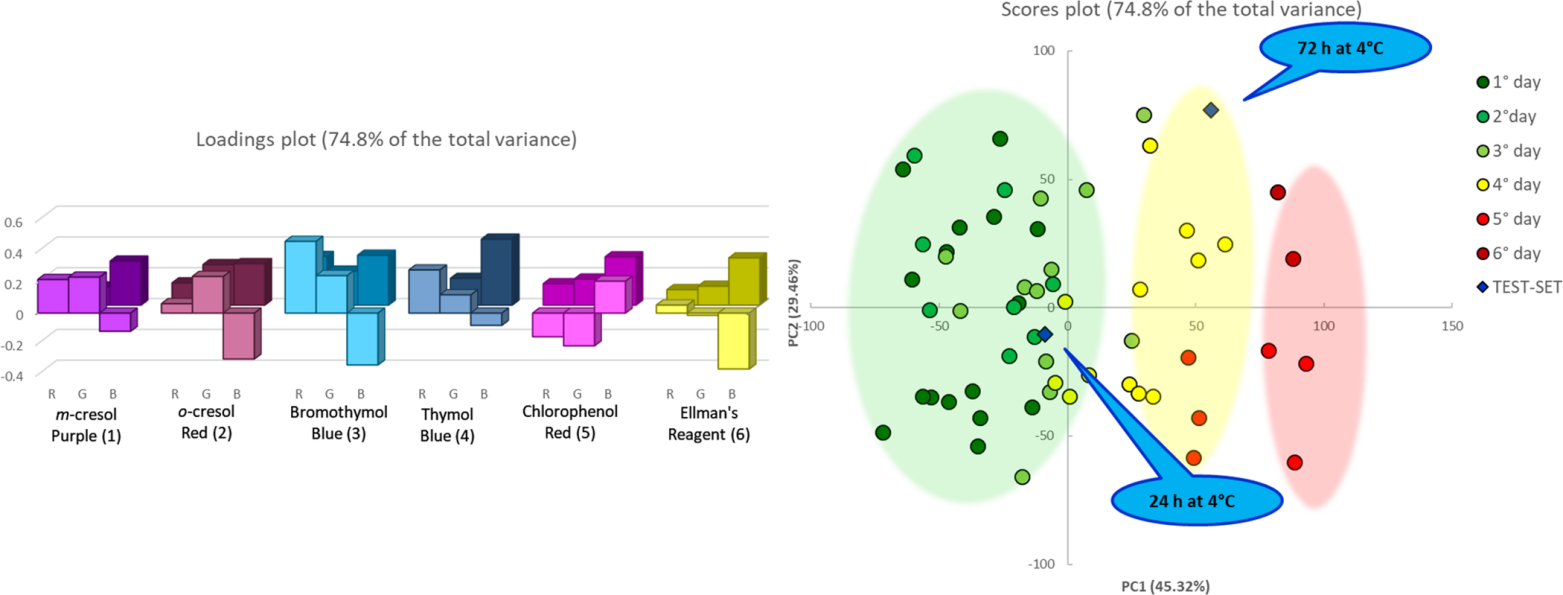
PCA model on six different samples of beast poultry meat for samples left in the fridge. On the right, the loading plot, in foreground values on the PCA1, in the background those on PCA2. On the right, the score plot. The green, yellow, and red shadow areas collect most of the samples defined safe, warning, and hazard, respectively.

The same PCA was also performed on the RBG evolution of the array in the samples kept in the fridge (six samples). The loadings and the scores plot on the two first components are shown in Figure 4.

In this case, comparing with the previous dataset, the separation is less clear. Here, the variance captured by the two first components is around 75%. The variability of the data set is higher, possibly because the more extended monitoring makes the difference between the samples manifest, but still good.

**Figure 5:**
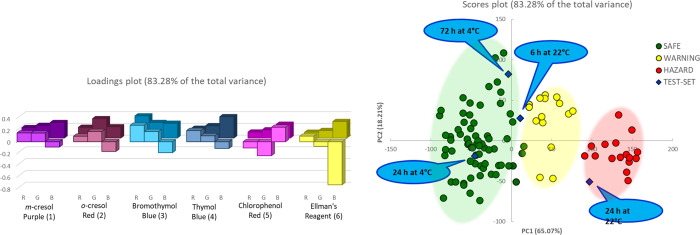
PCA model on the entire data set, considering the first two components that explain 80.3% of the total variance. On the left, the loading plot, in foreground values on the PCA1, in the background those on PCA2. On the right, the score plot. The blue bubbles are relative to external samples, see text. The green, yellow, and red shadow areas collect most of the samples defined safe, warning, and hazard, respectively.

Nonetheless, after observing that the degradation is the same for the two series of samples, we performed a PCA on the entire data set, now made by 11 samples, following the color of the array of six sensors described by three coordinates every single one. We simply assigned the categories SAFE, WARNING, and HAZARD, as estimated by the previous PCAs, which are labels, so not included in modeling. Figure 5 reports the PCA results, with loadings and scores plots on the first two components, which account for the 80.3% of the variance for the overall PCA.

They are not dissimilar from those of Figures 3 and 4. As for the analysis of separated samples, the first component accounts for the spoilage process. The samples in the first hours will occupy the left part of the score graph. As the spoilage process goes on, independently if samples are kept in or out of the fridge, the headspace atmosphere varies, provoking a reaction into the sensors, the dyes change their colors, and the samples move to the right part on the graph. The second component of PCA that accounts for 18.6% of the variance is related to sample variability, which seems to decrease in the last hazard step.

Moving along the score plot, from left to right, with different rates, the difference between the samples kept in or out of the fridge is evident. The conservation time is limited to 53 and 96 h, respectively, since after the decomposition, it is very evident, also without a sensor response.

It is worth noting that the loading plot is very similar indeed in the three cases, as expected from what was assessed above. Some dyes are more informative than others, as tested looking at the colors of the different devices as a function of time, in Figure 2. Using this array, on the CC solid support, for this type of protein substrate, the final choice for an in-field application on a large scale, which describes all the different degradation stages, could be limited to bromothymol blue (**3**), chlorophenol red (**5**), and Ellman’s reagent (**6**), the second fundamental to describe the evolution from SAFE to WARNING, first and the third from WARNING to HAZARD. (See below, in the section Future Developments.)

**Figure 6:**
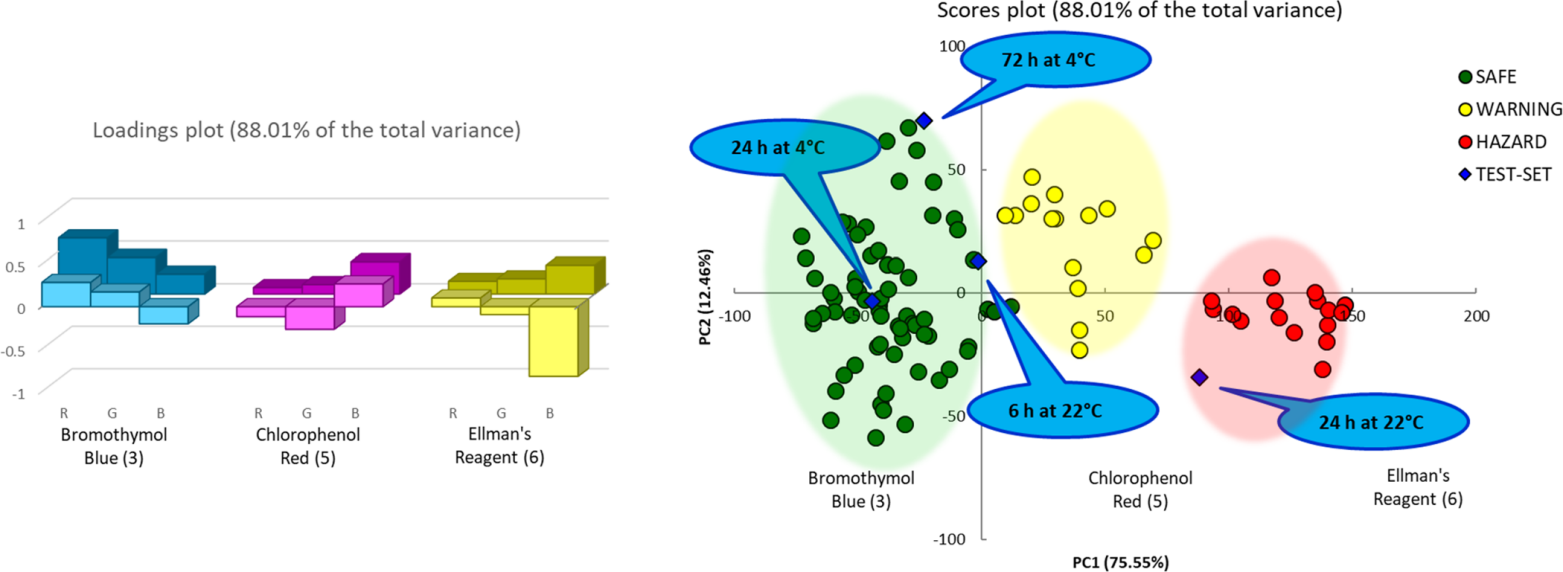
PCA model on the entire data set, considering only the most significant sensors, based on the first two components that explain 88.1% of the total variance. On the left, the loading plot, in foreground values on the PCA1, in the background those on PCA2. On the right, the scores plot. The blue bubbles are relative to external samples, see text. The green, yellow, and red shadow areas collect most of the samples defined safe, warning and hazard, respectively.

The PCA analysis on the same extended data set but limited to these three sensors is reported in Figure 6; the similar identification of groups is maintained, and the first two components capture 88% of the total variance.

Anyway, the setup made of three sensors not only cover the entire spoilage process but helps in making the decision about the fate of the poultry meat. Two sensors are pH indicators and cannot be in contrast. At worst, one of them could be in a color in between the two acid/base forms, and the color of the other one is of help. The thiols’ sensor works only in the presence of sulfur compounds, typical of the least phase and its yellowing confirms when the hazard step is approaching.

### PCA Model Validation

The attribution to the different spoilage steps sounds very nice, but it could be an artefact. As a first naive validation of the correct “classification”, we projected into this PCA model, built with all the samples, the RGB data of the array of four new trays, keeping two of them into and two out of the fridge as sort of blind samples. They are reported in the score figures with blue rhombuses, and they were always correctly attributed, either in the model with the six sensors as in the reduced to three. We understand, strictly speaking, classification needs other tools, but first here we want only to demonstrate that the color evolution is associated with the real spoilage.

As a further assessment, we directly analyzed the meat samples by instrumental analysis. We examined three samples, split in two subsamples for each stage, as reported in the Materials and Methods (Sample Validation and Validation Analysis). Further details on the analytical procedures can be found in the first chapter of the Supporting Information, while the results of the analyses on the meat are in Table 2.

**Table 2:**
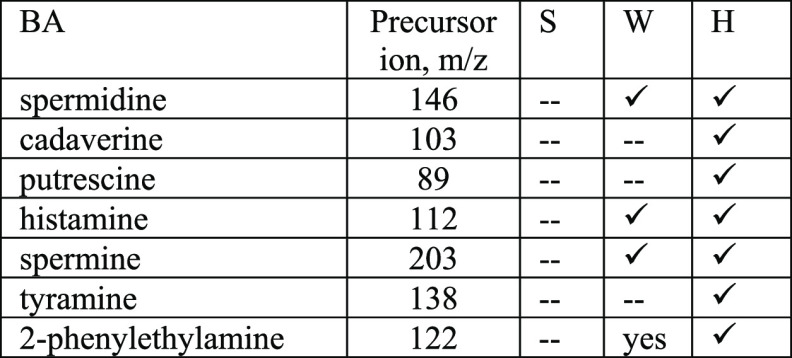
Identification of BA Performed through HPLC-ESI/MS Analysis on Different Samples of Poultry Meat Analyzed in Duplicate in Correspondence with the Three Degradation Steps Identified by PCA: S (Safe), W (Warning), and H (Hazard)

The results of the analyses on the meat samples show very clearly the developments of the BAs, present in the two last stages of the three degradation steps recognize by PCA. In the last one, all seven identified amines are present. Conversely, in samples belonging to the cluster defined as SAFE, no BAs were detected, while four of seven in the stage were defined as WARNING. The extraction gave identical results on two replicates.

This result represents a validation of the PCA model, meaning that clusters are not an artefact and that the colors associated with each step identify the meat condition, independently of the expiry date. The attribution of the stage is reliable, giving value to the idea of an implementation of the array into a label created and designed explicitly for this purpose.

We also wanted to analyze the composition of the atmosphere over the meat for samples at an identical degradation stage. The results of analyses obtained with the Headspace solid-phase microextraction (HSSPME), coupled with gas chromatography–mass spectrometry (GC/MS), performed on the headspace of the same samples are reported in Table 3. Alcohols, clearly visible ethanol, aldehydes, and acids, in particular acetic acid, are the characteristic component of the early stage atmosphere.

**Table 3:**
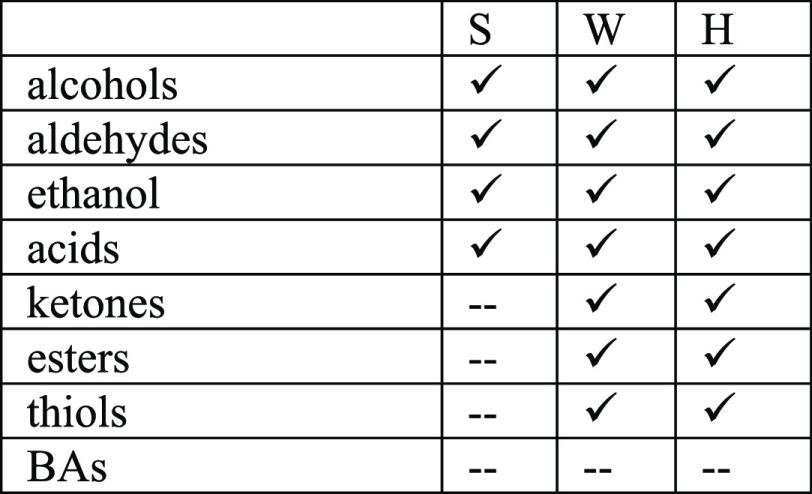
Identification of the Class of Substances Detected in the Headspace Performed through HPLC-ESI/MS Analysis on Different Samples of Poultry Meat Analyzed in Duplicate in Correspondence with the Three Degradation Steps Identified by PCA: S (Safe), W (Warning), and H (Hazard)

Ketones, esters, and thiols appear after but no amines, confirming our statement. Of these molecules, only sulfur compounds can be detected by our array, in the samples classified as WARNING and HAZARD.

What has never been underlined, at the authors’ knowledge, is that BAs do not appear in the headspace of the samples at the same degradation step when they are actually found inside the meat, neither in those belonging to the SAFE cluster nor in those of WARNING and HAZARD ones.

**Figure 7:**
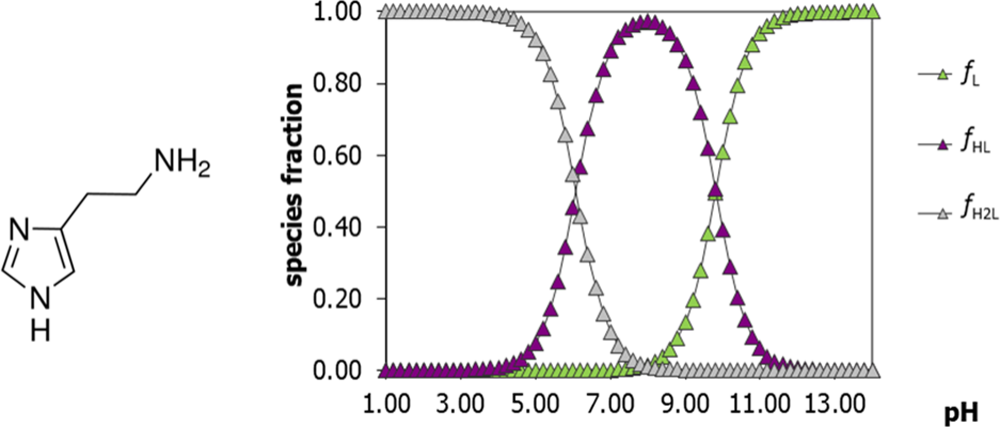
Speciation scheme of acid–base equilibria of histamine (on the left) in water solution-based protonation constants found in the literature,^27^ where L corresponds to the fully deprotonated form, HL to the monoprotonated, H_2_L to the fully protonated form.

The results fully justified the fact that the spots do not turn into their basic color, as expected in the early beginning of this research, and as always mentioned by the literature on these colorimetric sensors. Indeed, BAs must not be expected in the headspace, and looking at their log *K*_a_, it seems definitively odd, considering that the pH of meat, even in advanced spoilage state, never exceed neutral pH. As an example, the distribution diagram of histamine is reported based on log *K*_a_ values reported in the literature.^27^ For all BAs mentioned above, the diagrams of distribution species as a function of the pH are not so different, meaning that the deprotonated form, L (reported with green triangles in Figure 7, the only form that could be present over the meat), which corresponds to the structure reported in Figure 7 on the left, starts to be significantly produced above pH 9. It is well documented that the pH of the meat never reaches those value,^11,26,28^ and indeed BAs were never detected in VOCs,^3,29^ while sulfur-based substances are. Indeed, the most crucial role in the off-flavor of meat comes from the volatile sulfur compounds. Sensory data suggest that they are responsible, at least in part, for the putrid, sulfuryl odor, which becomes superimposed in an advanced stage of storage in the air on the earlier developing fruity odor,^25^ the only exception being the fish, which always “smells of fish” since an entirely different secretion system characterizes it.

### Sensitivity

Another aspect that was of concern was the sensitivity of the array, tailored around the most common tray of beast poultry meat present on the market. The experiments with decreasing meat fractions demonstrated that evolution is the same until a ratio of 150 g/500 cm^3^, corresponding to 50% of the reference mass. For lower ratios, the evolution for extreme times is still like that of the model, while at intermediate steps, the assignment is definitively unclear. This result means that, in these samples, the acid analytes, typical of the first part of the degradation, are not enough concentrated in the headspace to be revealed by the dyes with higher log *K*_a_. Opposite, a slight increase in the pH and the presence of volatile thiols are well detected even at the lowest fraction.

### Classification Attempt

This research was a preliminary test for the chameleon array for in-field application, and at this stage, experiments were never planned to perform classification. With the reduced data set, involving only the three final sensors, i.e., with a number of variables equal to 9 (three sensors described by three RGB indexes), we fulfill a basic requirement to have a number of objects per class at least equal to the number of variables and to be under the condition required for LDA, linear discriminant analysis. Still, it is not an optimal condition since the dispersion of the samples is definitively not equal (but there are not yet enough samples to move to QDA, quadratic discriminant analysis). As reference samples to be used as a training set, we choose those whose attribution to one of the classes was without doubts. Indeed, the authenticity cannot be assessed independently, as suggested by the literature,^30^ but the results of the attempt are shown in Table 4. In cross-validation, the prediction was satisfactory been of 100%.

**Table 4:** Results of the Classification Attempt on the Reduced Data Set with Nine Variables

confusion matrix in cross-validation			
	HAZARD	SAFE	WARNING
HAZARD	13	0	0
SAFE	0	22	0
WARNING	0	0	9

% Correct Predictions in Cross-Validation			
HAZARD	SAFE	WARNING	
100	100	100	

**Figure 8:**
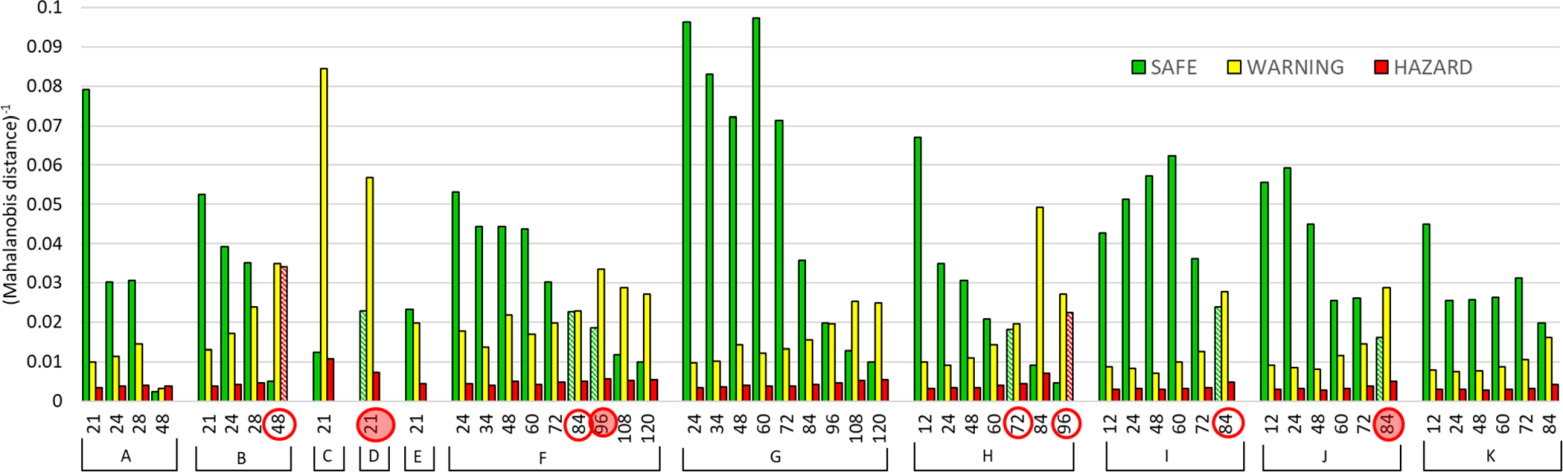
Results of the classification on 54 photos acquired on different poultry meat trays (from A to K) at increasing time (reported in hours on the *x* axis). On the *y* axis, the inverse of the Mahalanobis distance of each photo from each of the three classes. Samples with the wrong attribution are within red circles.

The set of values to test the classification model was made of 54 photos acquired on different poultry meat trays collected over time. Samples were correctly assigned in 85.2% of cases. The software CAT gives, as an output, the Mahalanobis distance from the three classes of each sample. In the graph of Figure 8, the reciprocal of the distance is reported for each sample of the test set, so for each sample, the highest bar represents the class closest to that sample, so the assigned one. The wrong attributions are highlighted with a red circle. Remembering that LDA gives a dichotomic answer (a sample is or in or out of the class) without uncertainty, it is worth noting that, in five cases on eight classified as wrong, the distance between the class attributed by the model is very close to that independently assigned, recognizable by the striped color. The three cases deeply far from the assigned class are highlighted with the red bubble with pink shadow.

When a final array will be developed, a deep classification model will be performed with the needed number of samples, training sample attribution independently assessed and possibly with other classification methods.

### Future Developments

**Figure 9:**
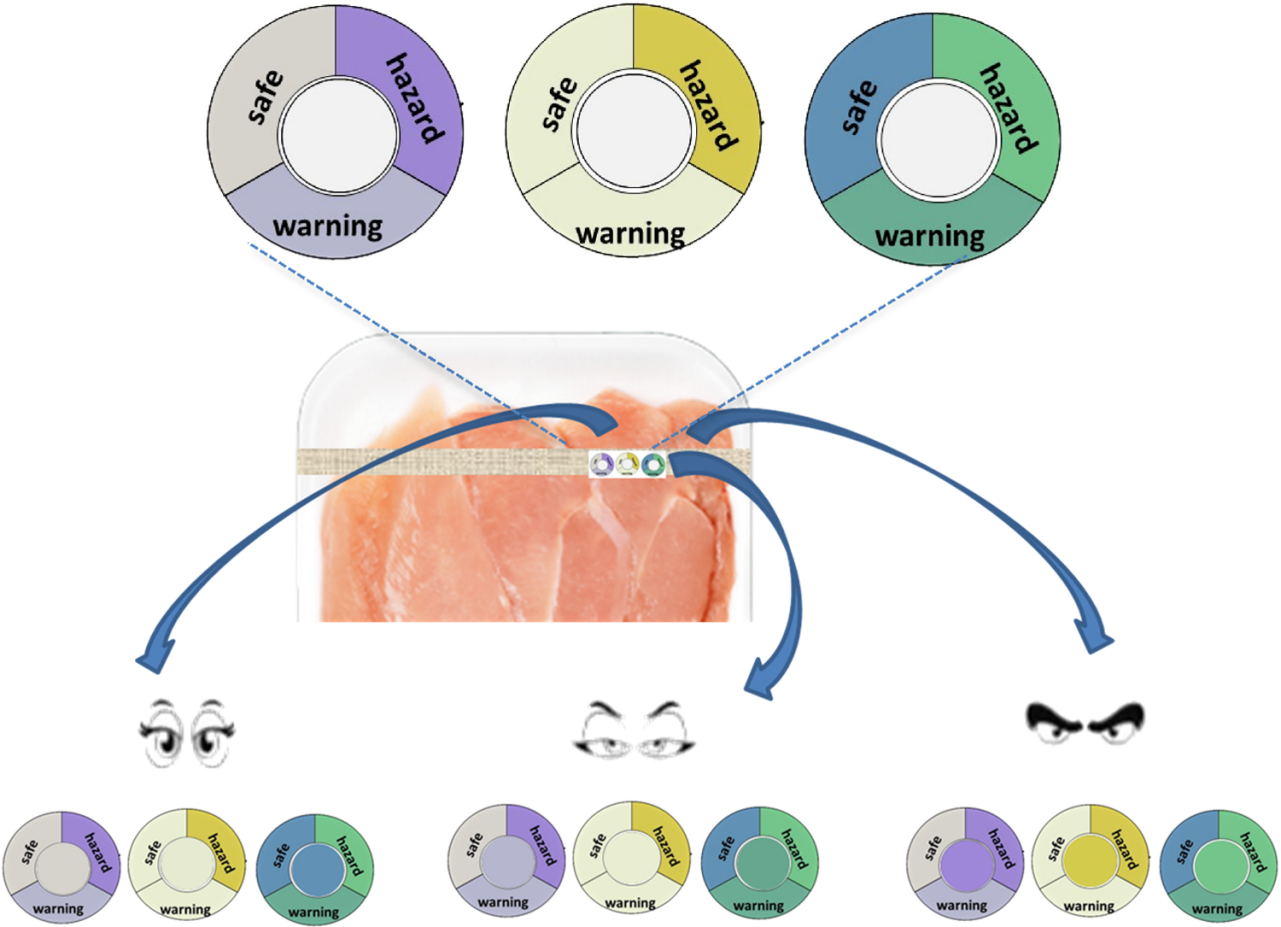
Possible prototype of the final array incorporated into the label. From left to right: chlorophenol red-CC@, Ellman’s CC@, and bromothymol blue-CC@. The reference colors of the three stages (SAFE, WARNING, and HAZARD) are printed in the crown that surrounds each sensor put in the center. The possible combinations on which basis a consumer decides the fate of the piece of meat is shown by different “eye expression”.

Based on what already underlined about the information carried by the different sensors of the array, we thought to a possible prototype of the intelligent label. An attempt of the concept is shown in Figure 9. On the upper part three crowns, with sectors printed with the average color of each step, surround each sensitive device, placed in the middle. The idea is covered by patents.^17,18^

The sensors are chlorophenol red (**5**), Ellman’s reagent (**6**), and bromothymol blue (**3**), whose colors change the most and allow to identify the univocally the correct degradation steps.

Comparing the colors of the spot with that in the sectors, by naked eyes, the consumer, in our idea, has precise information about the degradation step of that specific piece of meat, as shown in the lower part of Figure 9 and take his/her decision of what to do with that piece of meat to consume it as scheduled, consume it immediately with proper cooking, or throw it away.

A further point under study is the behavior of the array with other substrates, such as beef, pork, fish, but also milk. These attempts were already partially done, and preliminary results are promising. The evolution of colors accounts for the different compositions of protein food, which mainly affects the duration of the degradation step.

The future implementation of the array is presently under a patent,^17^ and a PCT was also deposited.^18^ They represent the development where the dyes are not anymore fixed on the CC but are covalently bound to a polymeric matrix. The idea is to have a more suitable material to be implemented into a label for industrial application.

## Supporting Information Available

An example of how we selected the proper acidification (or basification) of the most suitable form of a sensor; the case of bromothymol blue sensor (Figure 1S), evolution of colors in the case of replicate samples of poultry meat kept out of the fridge and in the fridge, (Figures 2S and 3S, respectively); details of the instrumental analysis on the meat samples belonging to three different degradation steps, chapter 1, and their chromatograms (Figure 4Sa, 4sB, 4Sc) (PDF)
