# Supplementary material for: Development of a Dye-Based Device to Assess the Poultry Meat Spoilage. Part II: Array on Act
Source: J Agric Food Chem. 2020 Oct 29;68(45):12710–8. doi: 10.1021/acs.jafc.0c03771 (PMC8015225; doi:10.1021/acs.jafc.0c03771)
Supplement: Supplementary file 1 — jf0c03771_si_001.pdf [file jf0c03771_si_001.pdf]

Supplementary section

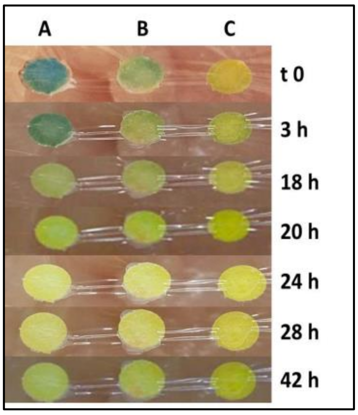

Figura 1S: Selection of the proper amount of acid to guarantee the sensor performance. Here the case of bromothymol blue sensor acidified with different amounts of acid.

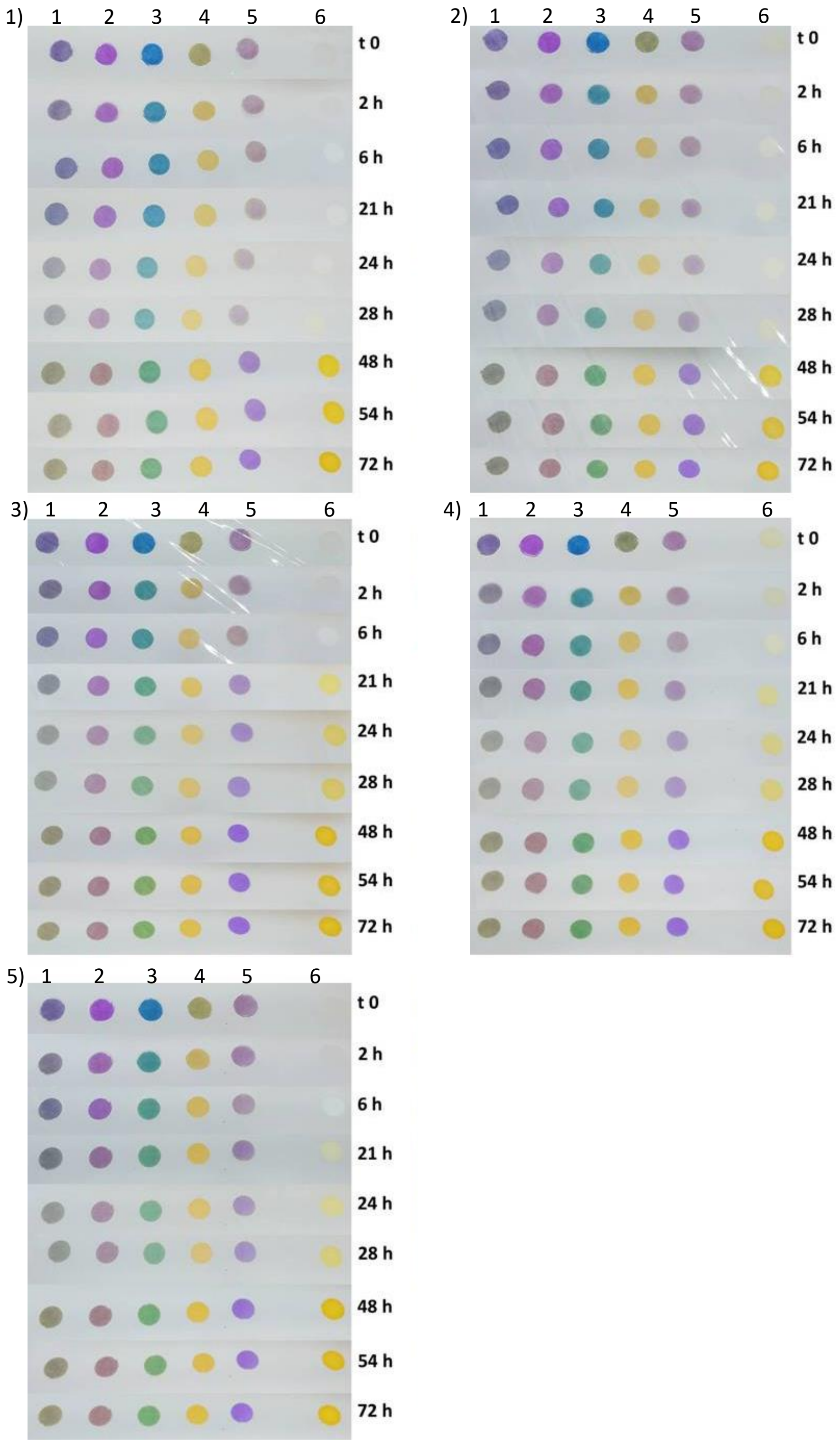

Figure 2S. The collage of the photos of the array over different samples as a function of the time, for the poultry meat out of the fridge.

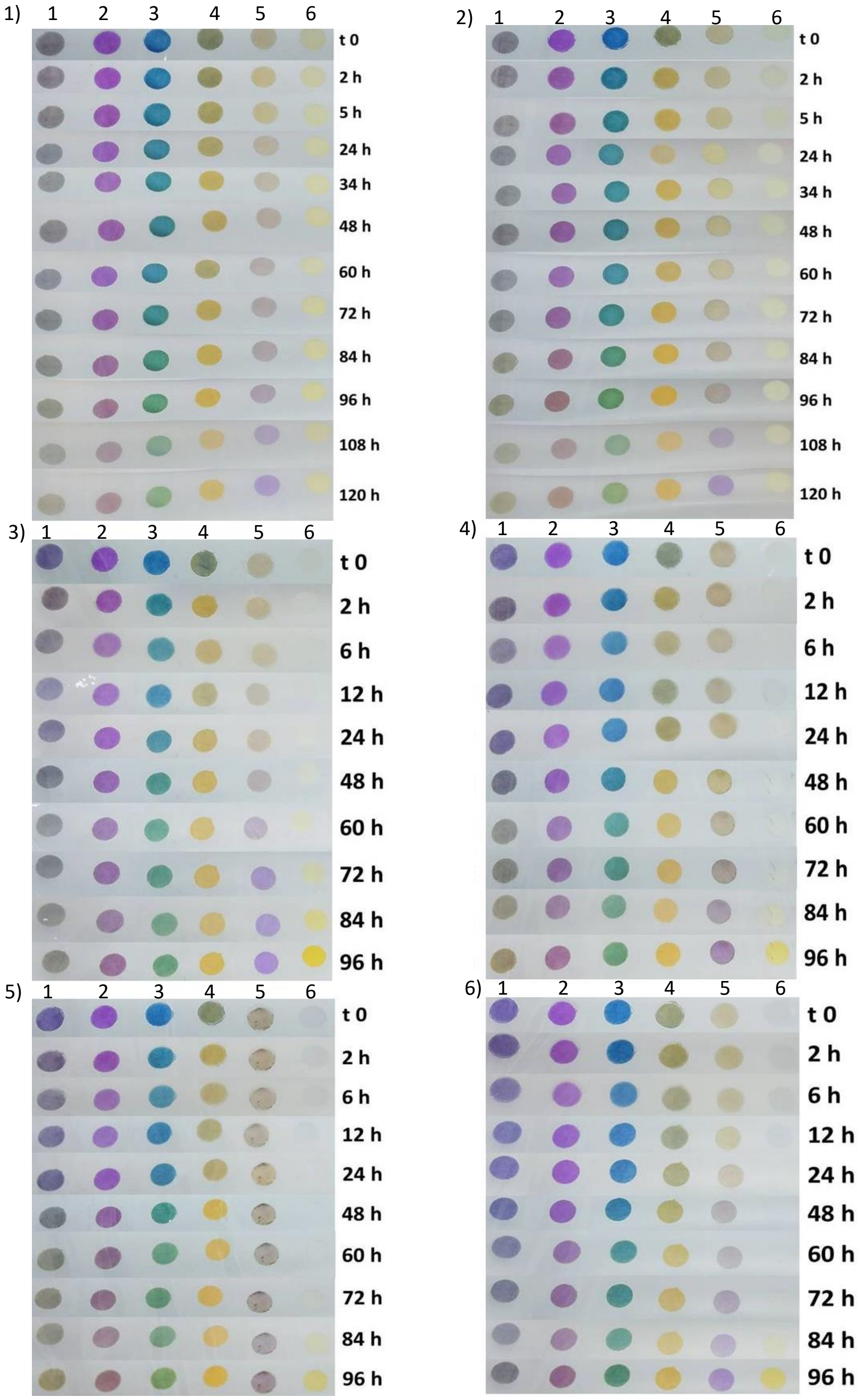

Figure 3S. The collage of the photos of the array over different samples as a function of the time, for the poultry meat in the fridge.

1. Details of the instrumental LC ESI/MS analysis

HPLC-ESI/MS analyses have been carried out on a Thermo Scientific LCQ FLEET system (LCQ FLEET ion trap mass spectrometer, Surveyor MS Pump/Autosampler/PDA Detector).
The separation of the biogenic amines (Ba) was achieved using a Synergi Fusion-RP-80A analytical column (150 × 2.0 mmI.D., particle size 4 µm) from Phenomenex. The mobile phase for HPLC-ESI/MS analysis was ammonium formate 5mM and formic acid in water (pH = 3.3) (A) and methanol (B), at a flow rate of 0.3 mL/min. The gradient program was: 0 min 30% B, 0–15 min 90% B, 15–20 min 30% B and 20–25 min 30% B. The injection volume was 20 µl. An Electro Spray Ionization (ESI) interface was used as an ion source, under positive ion conditions. The acquisition was performed both in Full Scan mode (mass range 50-2000 Da) and Dependent Scan mode. The Ion Spray voltage and Capillary voltage were set at 5 kV and 33 V in positive ion mode. The capillary temperature was 300°C. The data station utilized the Xcalibur MS Software Version 2.1.

In the following, HPLC-ESI/MS chromatograms acquired in MS full scan/extract ion of analyzed samples are reported, in the case of three different samples, defined as HAZARD, figure 4Sa, WARNING, figure 4Sb and SAFE figure 4Sc.

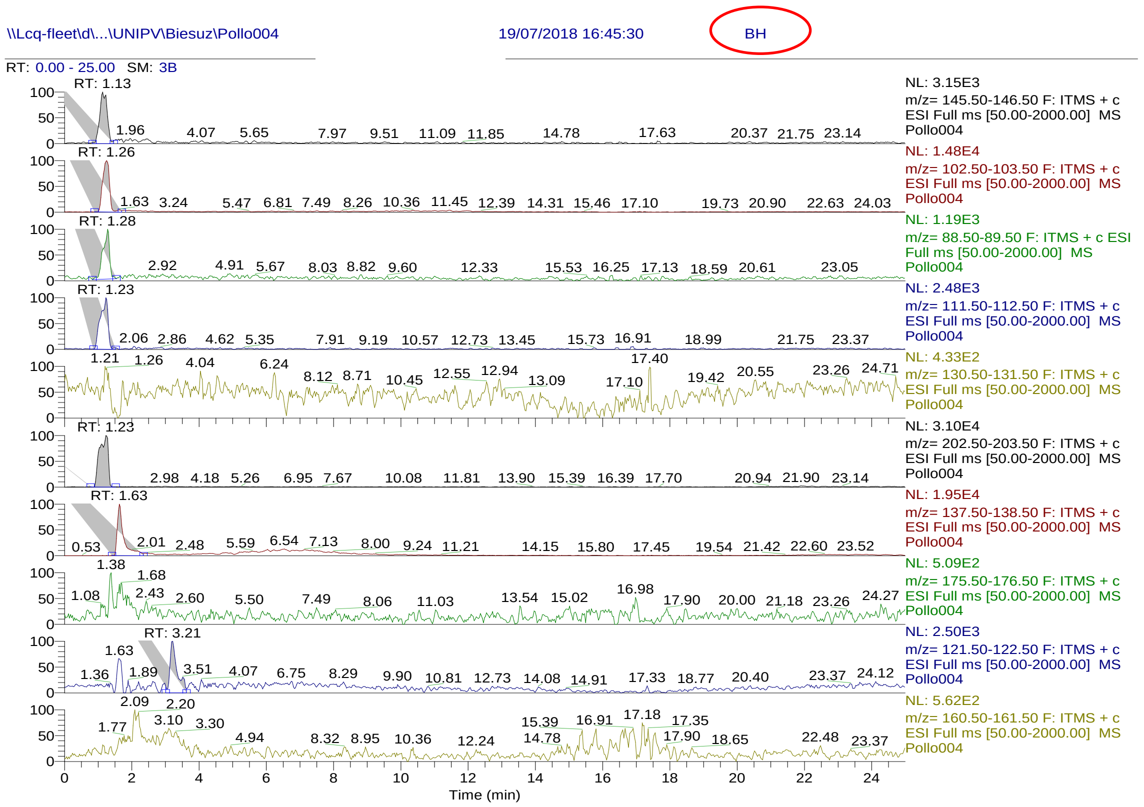

Figure 4S a - Chromatogram of the sample defined as HAZARD.

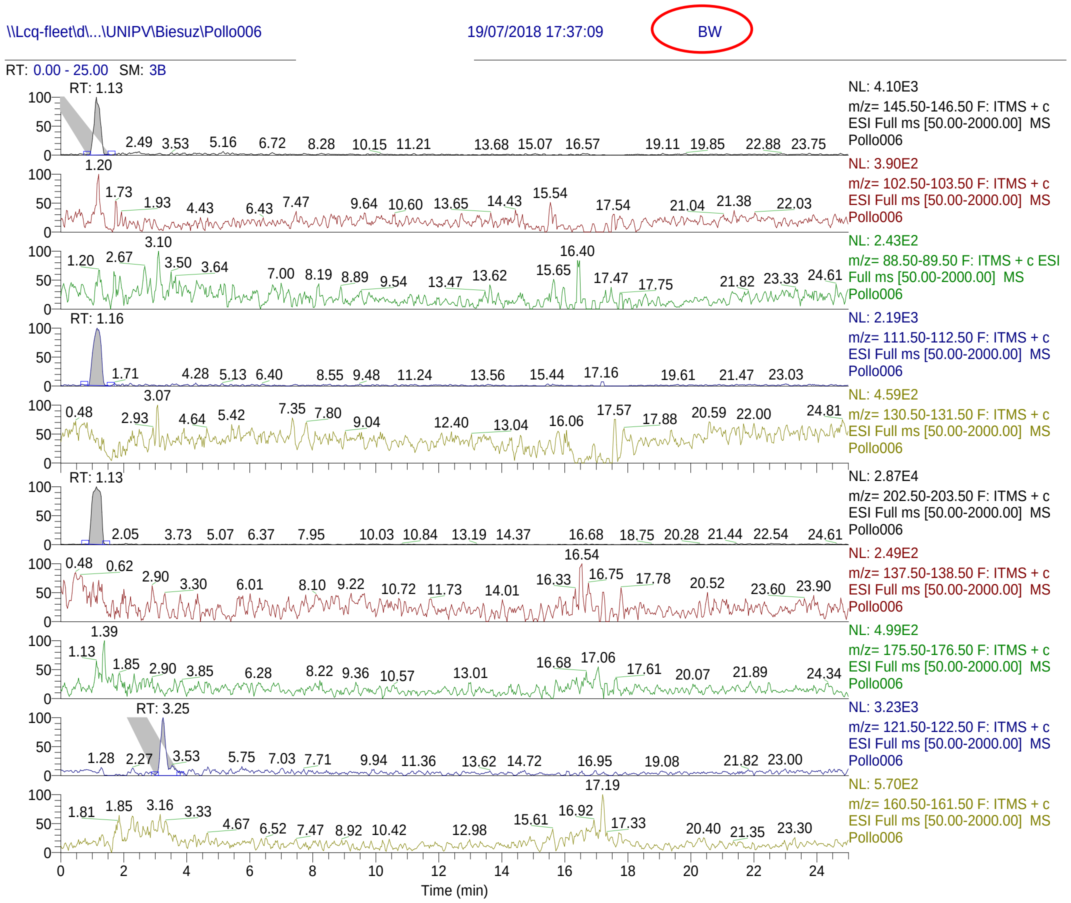

Figure 4S b - Chromatogram of the sample defined as WARNING.

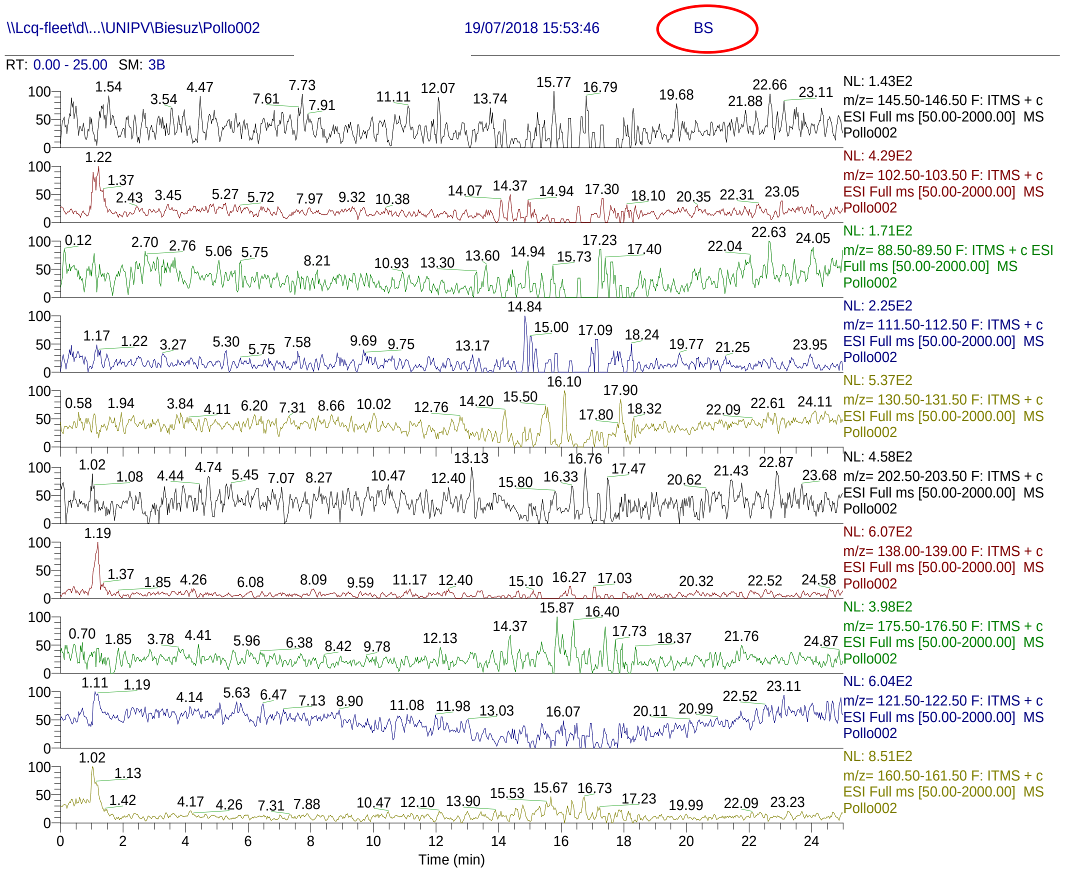

Figure 4S c - Chromatogram of the sample defined as SAFE.
